# *E2F1*-induced lncRNA, *EMSLR* regulates lncRNA *LncPRESS1*

**DOI:** 10.1038/s41598-022-06154-2

**Published:** 2022-02-15

**Authors:** Priyanka Priyanka, Madhur Sharma, Sanjeev Das, Sandeep Saxena

**Affiliations:** 1 National Institute of Immunology, Aruna Asaf Ali Marg, New Delhi, 110067 India; 2 JNU, New Delhi, India; 3 UDSC, New Delhi, India

**Keywords:** Cancer, Cell biology

## Abstract

*E2F1* induces hundreds of protein-coding genes influencing diverse signaling pathways but much less is known about its non-coding RNA targets. For identifying *E2F1*-dependent oncogenic long non-coding RNAs (lncRNAs), we carried out genome-wide transcriptome analysis and discovered an lncRNA, *EMSLR*, which is induced both in lung adenocarcinoma (LUAD) and lung squamous cell carcinoma (LUSC). *EMSLR* depletion blocks the cells in G1 phase and inhibits the clonogenic ability indicating that it is essential for the tumor-related phenotypes. We discovered that *EMSLR* represses the promoter activity of another lncRNA, *LncPRESS1*, which is located 6.9 kb upstream of *EMSLR* and they display an inverse expression pattern in lung cancer cell lines. Depletion of *C-MYC* results in downregulation of *EMSLR* and simultaneous upregulation of *EMSLR* target *LncPRESS1*, exemplifying how *C-MYC* and *E2F1* signal transduction pathways control the network of lncRNA genes to modulate cell proliferation and differentiation.

## Introduction

Long non-coding RNAs (lncRNAs) are RNA moieties that are more than 200 nucleotides long, posses a 5′ cap and 3′ poly A tail but lack a protein-coding open reading frame^1–3^. In the past long non-coding RNAs (lncRNAs) were thought to be transcriptional noise but subsequently functional mutations were mapped to the non-coding genome followed by discoveries of vital roles of lncRNAs in fundamental cellular processes and their association with a spectrum of diseases ranging from cancer to neurodegeneration^4,5^. LncRNAs are known to regulate gene expression by acting at both post-transcriptional and transcriptional levels. LncRNAs can influence expression at the post-transcriptional level in many ways such as by functioning as a competitive endogenous RNA (ceRNA) to regulate miRNA levels^6^. LncRNAs are also known to mediate post-transcriptional gene regulation by associating with RNA-binding proteins (RBPs) and regulating mRNA translation or stability^7^. LncRNA *LAST* cooperates with a RNA-binding protein known as CNBP to bind to the 5'UTR of cyclin D1 mRNA thus protecting it from nuclease degradation^8^. A *C-MYC* target lncRNA, known as *MYU*, is induced in colon cancer where it associates with hnRNP-K, a RNA-binding protein, to stabilize expression of cyclin dependent kinase 6 which results in higher proliferation and tumorigenicity^9^. LncRNAs mediate transcriptional regulation by functioning as an activator or repressor of the neighboring (cis-acting) or distant (trans-acting) genes. LncRNAs could act as signals, decoys, guides or scaffold mediating epigenetic regulation and chromatin remodeling^10^. For example lncRNA *KCNQ1OT1* acts as a signal by recruiting G9a histone methyltransferases and polycomb repressive complex 2 (PRC2; constituting of Ezh2, EED, SUZ1 and RbAp proteins) which mediate the gene-silencing-associated methylation^11,12^. Sequestering of transcription factor NF-YA by lncRNA *PANDA* exemplifies the decoy roles of lncRNAs^13^. LncRNAs can act as a ‘guide’ by recruiting either repressive or activating transcriptional complexes thus inducing chromatin change in *cis* in a cotranscriptional manner or in *trans* by binding to target DNA forming a triplex^14^. The most well-studied example of this function is lncRNA *XIST* which recruits the polycomb repressive complex 2 to mediate the chromosome-wide silencing of one of the two X-chromosomes in female mammals^15^. The guiding function is also well exemplified by lncRNA HOTAIR which promotes PRC2 to chromatin, leading to epigenetic gene silencing in HOXD loci^16,17^. LncRNAs can also function as ‘scaffolds’ when they serve as platforms upon which molecular components assemble, and in which case they would bind to multiple effector partners at the same time brings the effectors together in both time and space, for example LncRNA *ANRIL* acts as a modular scaffold and promotes the binding of WDR5 and HDAC3 complexes^18^. Another example is lncRNA HOTAIR which functions as a molecular scaffold when it binds PRC2 in the 5′domain and LSD1/CoREST/REST complex in the 3′domain^19^. It is now accepted that during carcinogenesis lncRNAs regulate basic cancer cell functions such as proliferation, apoptosis and invasion^9,20–25^. Comprehensive genome-wide analysis of more than 5,000 tumor samples across 13 cancer types have revealed lncRNAs alterations at the transcriptional, genomic and epigenetic levels^26^. These studies have reported that most dysregulated lncRNAs exhibit a tissue and cancer-type specific expression but there is a fraction of differentially regulated lncRNAs that are common across different cancer types. Despite the progress in understanding lncRNA function in human cancers, majority of lncRNAs have not been functionally evaluated. Further, recent sequencing studies have revealed hundreds of new uncharacterized lncRNAs and thus there is a need for functional characterization of the differentially expressed lncRNAs to establish their role in oncogenesis^27^.

*E2F1* transcription factor induces multitude of protein-coding genes involved in diverse cellular functions such as DNA replication, cell cycle and apoptosis, but only a few lncRNAs targets of *E2F1* have been functionally described^28^. These examples include chromatin-associated LncRNA *RP11-19E11* which is required for the proliferation of breast cancer cells^29^. Another lncRNA known as *ERIC* is activated by *E2Fs* whose inhibition increased *E2F1*-mediated apoptosis, implying that *E2F1* and *ERIC* constitute a negative feedback loop to modulate *E2F1* activity^30^. On the other hand lncRNA *RAD51-AS1*, which promotes cell cycle progression and inhibits apoptosis in epithelial ovarian cancer cells, is repressed by *E2F1*^31^. Thus, we are now beginning to comprehend the *E2F1* control of lncRNA expression but our understanding of the lncRNA targets of *E2F1* remains limited and the vast majority of lncRNAs have yet to be evaluated. In this study we have attempted to identify *E2F1*-dependent oncogenic lncRNAs. We have carried out transcriptome analysis of human cancers to identify the lncRNAs that are dysregulated in lung adenocarcinoma (LUAD) and lung squamous cell carcinoma (LUSC). We discovered that an lncRNA, *EMSLR*, which is induced both in LUAD and LUSC, is dependent on *E2F1* for its expression. We discovered that *EMSLR* represses another closely-located lncRNA known as *LncPRESS1*. Depletion of *EMSLR* demonstrates that it is an oncogenic lncRNA that mediates the aggressive phenotypes of cancer cells.

## Results

### Transcriptome analysis identifies *EMSLR*, an *E2F1*-dependent lncRNA that is upregulated in LUAD and LUSC

**Figure 1:**
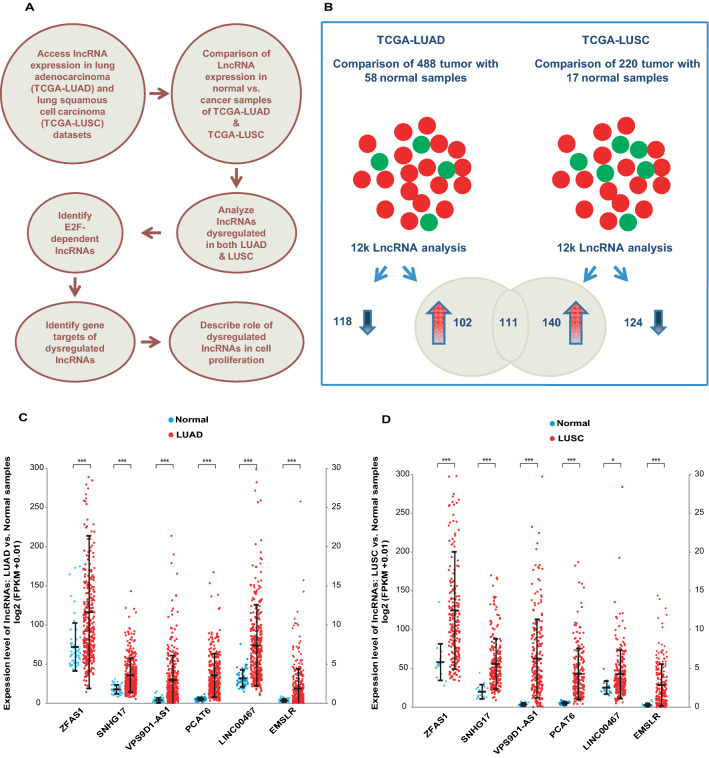
Transriptome analysis identifies *EMSLR*, an *E2F1*-dependent lncRNA that is upregulated in TCGA-LUAD and TCGA-LUSC datasets. (**A**) Strategy used for the identification of *E2F1*-dependent dysregulated lncRNAs in LUAD and LUSC, determination of their gene targets and their effect on cell proliferation and oncogenesis. (**B**) Comparison of transcriptional profiles identifies 111 lncRNAs upregulated in both LUAD and LUSC. The expression of around 12,000 lncRNAs was compared between 488 tumor and 58 normal samples of LUAD as well as 220 tumor and 17 normal samples of LUSC to identify upregulated (fold change > 2; upright arrow) or downregulated (fold change < 0.5; inverted arrow) lncRNAs. 111 lncRNAs that were upregulated in both cancers have been depicted by an intersecting Venn diagram. (**C**) From the 111 upregulated lncRNAs, 6 lncRNAs were selected which were previously reported to have effects on cell proliferation and oncogenesis. The plot displays the relative expression of the selected lncRNAs, namely *ZFAS1*, *SNHG17*, *VPS9D1-AS1*, *PCAT6*, *LINC00467* and *EMSLR* in 488 tumor and 58 normal samples of LUAD dataset. Each point refers to the levels of selected lncRNAs in one sample, whereas long and short horizontal bars represent the mean and S.D., respectively. Due to exceptionally high expression of *ZFAS1*, its log_2_ FPKM values has been plotted with respect to the left vertical axis (range from 0–300), while the other lncRNAs have been plotted with respect to the right vertical axis (range from 0–30). *p* values calculated using Student’s t test display that expression of the selected lncRNAs is significantly different in LUAD samples compared to their respective normal samples (*p < 0.05; **p < 0.01, ***p < 0.001). (**D**) Relative expression of the selected lncRNAs in 220 tumor and 17 normal samples of LUSC dataset. Details are same as part (**C**).

We followed a scheme for identifying common dysregulated lncRNAs as described in Fig. 1A. We obtained the lncRNAs expression data for normal and cancer samples of LUAD and LUSC datasets from the TANRIC data portal, which lists around 12,000 lncRNAs from the TCGA database (Fig. 1B)^32^. Using a transcriptome screen of analysis of LUAD dataset we have recently reported that an lncRNA *LINC02381* recruits RNA binding protein HuR to stabilize the 3′UTR of *HOXC10* mRNA^33^. In this present study we have compared the lncRNA deregulation observed in LUAD with LUSC as both are subtypes of the non-small cell lung cancer (NSCLC) and later in this study, we have followed up on the lncRNA leads by modulating lncRNA levels in two NSCLC cell lines, A549 and H1299. We compared lncRNA expressions between 488 tumor and 58 normal samples for LUAD, and between 220 tumor and 17 normal samples of LUSC to identify upregulated (fold change > 2) or downregulated (fold change < 0.5) lncRNAs in each cancer (Fig. 1B). By doing so, 213 and 118 lncRNAs were observed to be upregulated and downregulated, respectively in LUAD samples compared to the normal samples and for LUSC, 251 and 124 lncRNAs were upregulated and downregulated, respectively compared to the normal samples. 111 upregulated lncRNAs were common between the LUAD and LUSC datasets and since we were interested in identifying lncRNAs dysregulated in multiple cancers, we pursued this group of lncRNAs for further investigation. From the list of 111 upregulated lncRNAs, six lncRNAs were selected, namely *ZFAS1*, *SNHG17*, *VPS9D1-AS1*, *PCAT6*, *LINC00467* and *EMSLR* based on previous reports linking them to cell proliferation and oncogenesis^9,21–25^. Next, we plotted the expression levels of the selected lncRNAs in individual LUAD and LUSC samples where we observed that their levels were significantly increased in LUAD and LUSC samples (Fig. 1C–D).

**Figure 2:**
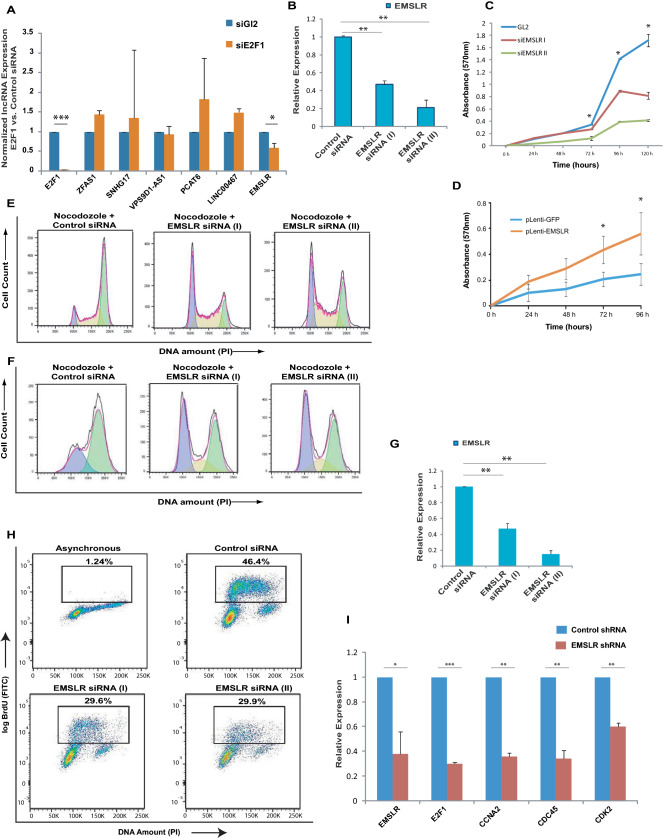
*EMSLR* depletion blocks the cell cycle progression. (**A**) Identification of *E2F1*-dependent lncRNA, *EMSLR*. A549 cells were transfected on three consecutive days with control *GL2* siRNA or *E2F1* siRNA and the levels of indicated lncRNAs were quantified by individual quantitative real-time PCR. The bar-graph indicates the levels of individual lncRNA in *E2F1* depleted samples relative to control *GL2* samples. Glyceraldehyde-3-phosphate dehydrogenase (*GAPDH*) was used as the endogenous control for normalization of lncRNA expression. (**B**) RNAi mediated depletion of *EMSLR*. A549 cells were transfected on three consecutive days with control *GL2* siRNA or siRNA targeting different regions of *EMSLR*: siRNA (I) and siRNA (II) followed by quantification of *EMSLR* levels by individual quantitative real-time PCR. *GAPDH* was used as the endogenous control for normalization of *EMSLR* expression. (**C**) Depletion of *EMSLR* reduces the rate of cell proliferation. MTT assay displaying the growth rates of *EMSLR*-depleted A549 cells as described in part (**B**) were seeded at equal numbers after transfection. The graph represents the growth rate with respect to control siRNA transfected cells at the indicated time after seeding the cells. (**D**) Overexpression of *EMSLR* enhances the rate of cell proliferation. A549 cells were infected with lentivirus expressing *EMSLR* or *GFP* followed by seeding of equal number of cells at 0 h and measurement of growth rates by MTT assay till 96 h. (**E**) Depletion of *EMSLR* leads to an accumulation of cells in the G1 phase. *EMSLR*-depleted A549 cells, as described in part (**B**) were obtained followed by treatment with nocodazole for 16 h and stained with propidium iodide (PI) for analysis of cell cycle distribution by flow cytometry. The percentage of cells in the G1 phase is significantly different in *EMSLR* siRNA samples compared to control *GL2* siRNA samples. (**F**–**G**) *EMSLR*-depleted H1299 cells were obtained by transfecting *EMSLR* siRNA (I) or siRNA (II) followed by treatment with nocodazole for 16 h and stained with propidium iodide (PI) for analysis of cell cycle distribution by flow cytometry. The efficiency of siRNA depletion of *EMSLR* in H1299 cells is shown in part (**G**). (**H**) Depletion of *EMSLR* impedes S phase progression. Flow cytometry of control *GL2* or *EMSLR* siRNA (I)-depleted A549 cells, as described in part (**B**), pulsed with BrdU followed by staining with FITC-conjugated anti-BrdU antibody along with propidium iodide. Dot plot displays BrdU incorporation (y-axis) and DNA content (x-axis) and inset shows the cells incorporating BrdU. The top-left panel (asynchronous) shows background FITC signal in the absence of anti-BrdU antibody. (**I**) *EMSLR* depletion results in downregulation of major cell-cycle related genes. A549 cells were transduced with lentiviral particles expressing either control shRNA or *EMSLR* shRNA followed by puromycin selection to obtain stable knockdown cells. The level of *EMSLR* and other cell-cycle related genes was quantified by individual quantitative real-time PCR. *GAPDH* was used as the endogenous control for normalization. The data is represented as mean of two independent experiments ± S.D. *p* values were calculated using two-tailed t-test (*p < 0.05; **p < 0.01, ***p < 0.001).

*E2F1*, a key transcription factor, mediates the expression of various genes involved in fundamental cellular functions mainly related to cell growth and proliferation is hyperactive in most human cancers including LUAD and LUSC^34–37^. Though *E2F1* transcription factor is known to induce hundreds of protein-coding target genes, few lncRNA targets of *E2F1* are known. In this study, we wanted to identify *E2F1*-dependent oncogenic lncRNAs so, we transfected A549, an aggressive lung adenocarcinoma cell line, with *E2F1* siRNA and evaluated the levels of the six shortlisted lncRNAs (Fig. 2A). We observed that most of the lncRNAs did not show decrease in expression after *E2F1* depletion but we observed that lncRNA *EMSLR* was significantly downregulated after *E2F1* depletion. In this study we have pursued the role of lncRNA *EMSLR* in cell proliferation and oncogenesis.

### *EMSLR* depletion blocks the cell cycle progression

In order to study the effect of *EMSLR* depletion, we carried out RNAi mediated depletion in A549 lung adenocarcinoma cells by transfecting siRNAs that target different regions of *EMSLR* and we observed that both the siRNAs significantly depleted the endogenous *EMSLR* (Fig. 2B). We performed MTT proliferation assay to evaluate the growth rates of *EMSLR*-depleted cells where we observed that *EMSLR* depletion significantly reduced the rate of cell proliferation (Fig. 2C). We also performed MTT proliferation assay to evaluate the growth rates of *EMSLR*-overexpressing cells where we observed that *EMSLR* overexpression increased the rate of cell proliferation demonstrating that *EMSLR* levels affects cell growth (Fig. 2D). To ascertain if *EMSLR* depletion leads to a G1 accumulation, *EMSLR* depleted A549 cells were treated with nocodazole to block the cells in G2/M phase, before evaluating the cell cycle distribution by flow cytometry. Nocodazole treatment reduced the G1 phase population of control cells by blocking the majority of cell population in the G2/M phase however the percentage of G1 phase population remains significantly higher in *EMSLR* depleted cells, thus demonstrating *EMSLR* deprived cells were arrested in G1 phase of cell cycle (Fig. 2E). We also carried out *EMSLR* depletion in H1299, another non-small cell lung cancer (NSCLC) cell line, and observed a similar G1 block demonstrating that the effect of EMSR is not cell line specific (Fig. 2F–G). We next evaluated the rate of DNA synthesis by measuring the incorporation of nucleoside analog, BrdU, using flow cytometry assay. We observed that a significant decrease in BrdU incorporation in *EMSLR* depleted cells as compared to control cells indicating that *EMSLR* depletion impedes S phase progression (Fig. 2H). We assayed if the effect of siRNA mediated *EMSLR* depletion can be rescued by exogenous expression of *EMSLR*. However, we observed that the exogenous expression could not significantly increase *EMSLR* levels in the presence of *EMSLR* siRNA, making it difficult to interpret the effect on cellular phenotypes. In order to understand the reason for the cell cycle block we assayed the expression of major cell cycle related genes. We transduced A549 cells with lentiviral particles expressing shRNA against *EMSLR* and obtained stable knockdown cells, which resulted in a significant decrease in *EMSLR* levels. We noted that *E2F1* transcription factor is downregulated after *EMSLR* deletion and thus it seems that *E2F1* and *EMSLR* are in a positive auto-feedback loop (Fig. 2A and I). A recent study has demonstrated that *EMSLR* maintains the level of *E2F1* by associating with a RNA-binding protein called RALY^23^. Concomitant with *E2F1* decrease there were the downregulation of the major cell cycle genes such as cyclin A2 (CCNA2), CDC45 and Cdk2 (Fig. 2I). Thus, the depletion of *EMSLR* results in downregulation of cell cycle activators resulting in a cell cycle arrest.

### *EMSLR* depletion inhibits the tumor-related phenotypes

**Figure 3:**
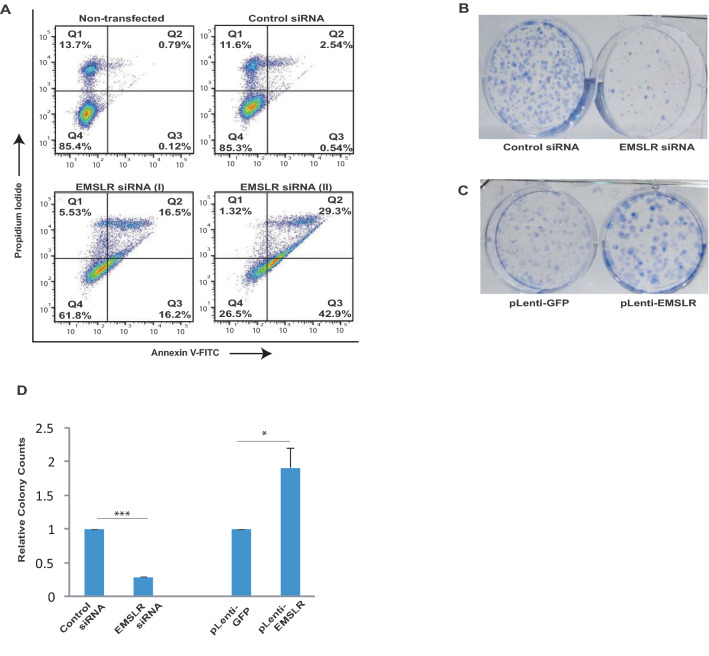
*EMSLR* depletion inhibits the tumor-related phenotypes of LUAD cells. (**A**) Depletion of *EMSLR* induces cell death in A549 cells. Flow cytometry of control *GL2* or *EMSLR*-depleted A549 cells, obtained by transfecting *EMSLR* siRNA (I) or siRNA (II), stained with FITC-conjugated anti-Annexin V antibody along with propidium iodide. Dot plot displays PI (y-axis) and Annexin V (x-axis) incorporation. Criteria were set to distinguish between viable (bottom left), early apoptotic (bottom right) and late apoptotic (top right) cells. (**B**–**D**) Clonogenic assay to evaluate the effect of *EMSLR* manipulation on A549 cells. **(B) ***EMSLR* siRNA (I)-depleted A549 cells as described in Fig. 2B were allowed to grow for 12 days, stained with crystal violet and the colonies were counted. (**C**) A549 cells were infected with lentivirus expressing *EMSLR* and grown for 12 days, followed by staining with crystal violet and the colonies were counted. Quantification of parts (**B**) and (**C**) is shown in part (**D**)*.* The data is represented as mean of two independent experiments ± S.D. *p* values were calculated using two-tailed t-test (*p < 0.05; **p < 0.01, ***p < 0.001).

In this study, *EMSLR* has been discovered from a screen to identify upregulated lncRNAs in human cancers and we wanted to evaluate if depleting *EMSLR* inhibits the tumor-associated phenotypes. Depletion of oncogenes such as *C-MYC* is known to induce apoptosis in cancer cells and thus we wanted to evaluate if *EMSLR* depletion also results in apoptotic death^38,39^. *EMSLR*-depleted cells were stained with FITC-conjugated anti-annexin V antibody along with propidium iodide (PI) and we observed that there was an increase in PI-negative, annexin V-positive cells which indicates early apoptosis, as well as PI-positive, annexin V-positive double stained cells which indicates late apoptosis (Fig. 3A). Next, we determined the clonogenic ability after *EMSLR*-depleted A549 cells which demonstrated that the depletion of *EMSLR* led to a significant reduction in the number of colonies formed (Fig. 3B and D). Colony forming ability was also determined in A549 cells infected with lentiviral vector expressing *EMSLR* which showed a mild increase in colony counts (Fig. 3C and D). Thus, we conclude that lncRNA *EMSLR* is associated with oncogenic phenotypes in cancer cells.

### *EMSLR* represses a closely located lncRNA, *LncPRESS1*

**Figure 4:**
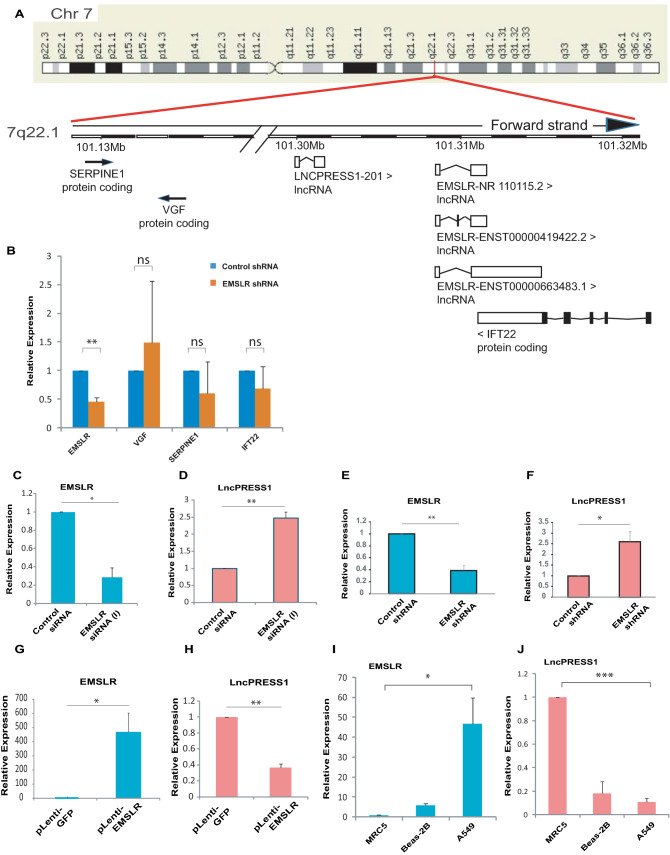
*EMSLR* represses a closely located lncRNA, *LncPRESS1***.** (**A**) G-banded ideogram representing human chromosome 7, showing the cytogentic location of *LncPRESS1*, *EMSLR*, *IFT22*, *SERPINE1* and *VGF* genes at the 7q22.1 cytogenetic band. The genomic coordinates of *LncPRESS1* gene are 101,299,613..101,301,346 while the *EMSLR* gene lies within 101,308,270..101,314,800 nt as per Genome Reference Consortium Human Build 38 patch release 13 (GRCh38.p13). Three alternatively spliced variants of *EMSLR* have been identified, namely NR 110115.2, ENST00000419422.2 and ENST00000663483.1. NR 110115.2 variant has been characterized as the functional *EMSLR* in a previous study^23^. The *LncPRESS1* and *EMSLR* gene are expressed in the same direction from the "forward" strand of chromosome 7. The *IFT22* gene which lies within the complement strand from 101,310,914..101,321,812 has a partial overlap with *EMSLR* gene. Also shown in the discontinuous ideogram are the locations of *SERPINE1* (101,127,104..101,139,247) and *VGF* (complement: 101,162,509..101,169,956) genes*.* (**B**) Effect of *EMSLR* depletion on the expression of the genes located in its vicinity. A549 cells were transduced with lentiviral particles expressing either control shRNA or *EMSLR* shRNA followed by puromycin selection to obtain stable knockdown cells and the levels of indicated genes were quantified by individual quantitative real-time PCR. (**C**–**D**) Depletion of *EMSLR* leads to upregulation of lncRNA *LncPRESS1*. A549 cells were transfected with control *GL2* or *EMSLR* siRNA (I) followed by quantification of *EMSLR* and *LncPRESS1* levels by individual quantitative real-time PCR. (**E**–**F**) Depletion of *EMSLR* by shRNA that targets a different region in *EMSLR* compared to *EMSLR* siRNA (I) or *EMSLR* siRNA (II) leads to upregulation of lncRNA *LncPRESS1*. A549 cells were transduced with lentiviral particles expressing either control shRNA or *EMSLR* shRNA followed by puromycin selection to obtain stable knockdown cells and quantification of *EMSLR* and *LncPRESS1* levels by individual quantitative real-time PCR. (**G**–**H**) Ectopic expression of *EMSLR* represses endogenous *LncPRESS1* expression. A549 cells were infected with lentiviral expressing *EMSLR* followed by quantification of *EMSLR* and *LncPRESS1* levels by individual quantitative real-time PCR. The data of part (**B**–**H**) is from mean of two independent experiments ± S.D. *GAPDH* was used as the endogenous control for normalization. (**I**–**J**) Relative expression of *LncPRESS1* and *EMSLR* in different lung cell lines with respect to MRC-5, a human lung fibroblast cell line derived from normal lung tissue which is used as a control for non small cell lung cancer^40^. The quantification of *EMSLR* and *LncPRESS1* levels in different cell lines was carried out by individual quantitative real-time PCR and normalized with 18 s RNA expression from two technical replicates. *p* values were calculated using two-tailed t-test (*p < 0.05; **p < 0.01, ***p < 0.001, ns, non-significant).

It is known that lncRNAs can regulate the expression of neighboring genes. *EMSLR* is expressed from the 7q22.1 cytogenetic band. Examination of the genomic locus from where *EMSLR* is expressed displays that another lncRNA known as *LncPRESS1* is located around 6.9 kb upstream of *EMSLR* (Fig. 4A). Apart from *LncPRESS1*, other protein coding genes located within 150 kb of *EMSLR* includes *VGF*, *SERPINE1*, and *IFT22*. We assayed the effect of *EMSLR* depletion on the expression of protein coding genes and observed that their expression was not significantly altered (Fig. 4B). We were interested in discerning the effect of *EMSLR* expression on the neighboring lncRNA *LncPRESS1* and thus, we transfected A549 cells with control or *EMSLR* siRNAs and evaluated the effect on *LncPRESS1* expression. We observed that *LncPRESS1* was upregulated after siRNA depletion of *EMSLR* (Fig. 4C–D). To further rule out non-specific effects, *EMSLR* depletion was carried out by shRNA that targets a different region in *EMSLR* compared to *EMSLR* siRNA (I) or *EMSLR* siRNA (II) (Fig. 4E–F). We observed that *EMSLR* shRNA-mediated depletion also leads to upregulation of lncRNA *LncPRESS1.*

Overexpression of *EMSLR* with a lentiviral vector led to a significant decrease in the levels of *LncPRESS1*, exemplifying that *EMSLR* represses *LncPRESS1* (Fig. 4G–H). Having identified *LncPRESS1* as a target of *EMSLR*, we assayed the expression pattern of *EMSLR* and *LncPRESS1* in cell lines displaying varying degrees of tumor-related phenotypes: (1) MRC-5, a human lung fibroblast cell line derived from normal lung tissue which is used as a control for non small cell lung cancer; (2) BEAS-2B, a non-tumorigenic lung epithelial cell line and (3) A549, an aggressive lung adenocarcinoma cell line^40^. We noted that the expression of *EMSLR* was significantly higher in aggressive cell line, A549 in comparison to lung fibroblast, MRC-5 (Fig. 4I). Evaluation of *LncPRESS1* transcript levels revealed that it is expressed at significantly higher levels in cell lines where *EMSLR* transcript levels are low exemplifying that these two lncRNAs display an inverse expression pattern (Fig. 4J). High levels of *EMSLR* coinciding with the low levels of *LncPRESS1* indicate that *EMSLR* targets *LncPRESS1* in transformed cells.

### *EMSLR* mediates the transcriptional repression of *LncPRESS1*

**Figure 5:**
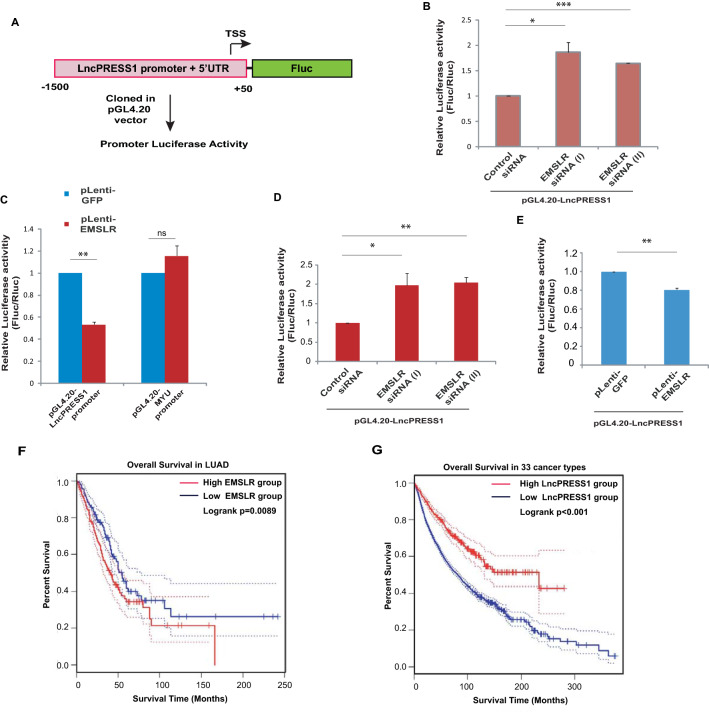
*EMSLR* mediates the transcriptional repression of *LncPRESS1*. (**A**) Schematic representation of the reporter plasmid containing the human *LncPRESS1* upstream region: The *LncPRESS1* promoter and 5′UTR region spanning − 1500 bp to + 50 bp with respect to transcriptional start site (TSS) was used to drive expression of the firefly luciferase gene (Fluc) in promoterless pGL4.20 vector (promega). (**B**) Depletion of *EMSLR*, by transfecting *EMSLR* siRNA (I) or siRNA (II), relieves the repression of *LncPRESS1* promoter in A549 cells. The pGL4.20 vector containing *LncPRESS1* promoter was transfected into control or *EMSLR*-depleted A549 cells together with a renilla luciferase (pRL-TK) reporter vector and both luciferase activities were measured after 24 h. The relative luciferase activity in each sample is expressed as a ratio of firefly to renilla luminescence. (**C**) A549 cells were infected with lentivirus expressing *EMSLR* followed selection with puromycin to obtain stable cells. The pGL4.20 vector containing *LncPRESS1* promoter was transfected into control or *EMSLR*-expressing stable A549 cells together with a renilla luciferase (pRL-TK) reporter vector and both luciferase activities were measured after 24 h. The relative luciferase activity in each sample is expressed as a ratio of firefly to renilla luminescence. (**D**) Depletion of *EMSLR*, by transfecting *EMSLR* siRNA (I) or siRNA (II), relieves the repression of *LncPRESS1* promoter in H1299 cells. The pGL4.20 vector containing *LncPRESS1* promoter was transfected into control or *EMSLR*-depleted H1299 cells together with a renilla luciferase (pRL-TK) reporter vector and both luciferase activities were measured after 24 h. The relative luciferase activity in each sample is expressed as a ratio of firefly to renilla luminescence. (**E**) H1299 cells were infected with lentivirus expressing GFP or *EMSLR* followed by selection with puromycin to obtain stable cells. The pGL4.20 vector containing *LncPRESS1* promoter was transfected into GFP or *EMSLR*-expressing stable H1299 cells together with a renilla luciferase (pRL-TK) reporter vector and both luciferase activities were measured after 24 h. The relative luciferase activity in each sample is expressed as a ratio of firefly to renilla luminescence. (**F**) Kaplan–Meier estimates of the survival of patients with low or high levels of expression of *EMSLR* in LUAD. The expression and survival information was downloaded from GEPIA (Gene Expression Profiling Interactive Analysis) platform. (**G**) Kaplan–Meier estimates of the survival of patients with low or high levels of expression of *LncPRESS1*. The sample size of LUAD was insufficient for correlating survival probability with *LncPRESS1* expression. Thus the survival probability was calculated in a combination of samples from all 33 cancers available at GEPIA with a high group cutoff of 10% and low group cutoff of 90%. The data is represented as mean of two independent experiments ± S.D. p values were calculated using two-tailed t-test (*p < 0.05; **p < 0.01, ***p < 0.001, ns, non-significant).

One of the ways by which lncRNAs influence expression of genes close to their locus is by modulating the promoter activity of the target genes. We analyzed the *EMSLR* and *LncPRESS1* promoter sequences by the LongTarget program which predicts the presence of triplex formation oligonucleotides (TFO) of *EMSLR* and their triplex targeting sites (TTS) within *LncPRESS1* promoter based on Hoogsteen and reverse Hoogsteen base-pairing rule (Table 1)^41^. In order to experimentally test whether *EMSLR* alters the promoter activity of *LncPRESS1*, we analyzed the effect of *EMSLR* depletion on the activity of luciferase gene driven by the *LncPRESS1* promoter and 5′UTR region spanning from − 1,500 bp to + 50 bp with respect to transcriptional start site (TSS) (Fig. 5A). We observed that depletion of *EMSLR* in A549 cells led to an upregulation of the luciferase activity driven from *LncPRESS1* promoter. Thus, it seems that *EMSLR* depletion led to de-repression of *LncPRESS1* promoter (Fig. 5B). On the other hand, ectopic expression of *EMSLR* with a lentiviral vector led to downregulation of *LncPRESS1* promoter activity (Fig. 5C). *EMSLR* overexpression did not alter the promoter activity of another lncRNA, MYU proving that *EMSLR* specifically mediates the transcriptional repression of *LncPRESS1*. We also assayed the effect of *EMSLR* on the activity of *LncPRESS1* promoter in H1299 cells and observed a similar transcriptional repression of *LncPRESS1* promoter demonstrating that the effect of EMSR on *LncPRESS1* promoter is not cell line specific (Fig. 5D–E).

**Table 1:** The LongTarget program prediction of the triplex formation oligonucleotides (TFO) of *EMSLR* and their triplex targeting sites (TTS) within *LncPRESS1* promoter based on Hoogsteen and reverse Hoogsteen base-pairing rule.

*EMSLR* region forming TFO	*LncPRESS1* promoter region at Chr 7q22.1	*EMSLR* TFO_sequence	*LncPRESS1* TTS_sequence
741–801	101,299,363–101,299,425	TGGGTTAATTTTTTTTTTTTTTTTTTTTTTGAGATGGAGTCTCGCTCTGTCGCCCAGGCTG	AGAAAGAAAGAAAAAGAAAAGAACAACGCGGCTCTGCGAGTACTGACACAAATCCCCAGCATA
745–801	101,299,368–101,299,425	TTAATTTTTTTTTTTTTTTTTTTTTTGAGATGGAGTCTCGCTCTGTCGCCCAGGCTG	GAAAGAAAAAGAAAAGAACAACGCGGCTCTGCGAGTACTGACACAAATCCCCAGCATA
745–795	101,299,370–101,299,420	TTAATTTTTTTTTTTTTTTTTTTTTTGAGATGGAGTCTCGCTCTGTCGCCC	AAGAAAAAGAAAAGAACAACGCGGCTCTGCGAGTACTGACACAAATCCCCA

### Survival analysis of patients with respect to *EMSLR* and *LncPRESS1* expression

Next, we correlated the *EMSLR* expression with survival information obtained from the GEPIA (Gene Expression Profiling Interactive Analysis) platform. Kaplan–Meier analysis showed that high levels of *EMSLR* are associated with low survival probability (Fig. 5F). However, Kaplan–Meier estimates of the survival of patients with low or high levels of expression of *LncPRESS1* could not be calculated for LUAD samples as the sample size of LUAD was insufficient for correlating survival probability with *LncPRESS1* expression. Thus, we calculated the survival probability with low or high levels of expression of *LncPRESS1* in a combination of samples from all 33 cancers available at GEPIA. Kaplan–Meier analysis showed that high levels of *LncPRESS1* are associated with high survival probability (Fig. 5G). Thus, patients with high levels of *LncPRESS1* exhibited an inverse pattern of survival as compared to patients with high levels of *EMSLR*. The clinical data analysis of the expression levels and survival probability suggests that *LncPRESS1* has a role in oncogenesis that is contrary to *EMSLR*.

### The transcriptional repression of *LncPRESS1* mediated by *EMSLR* is dependent on DNA Methyltransferase 1

**Figure 6:**
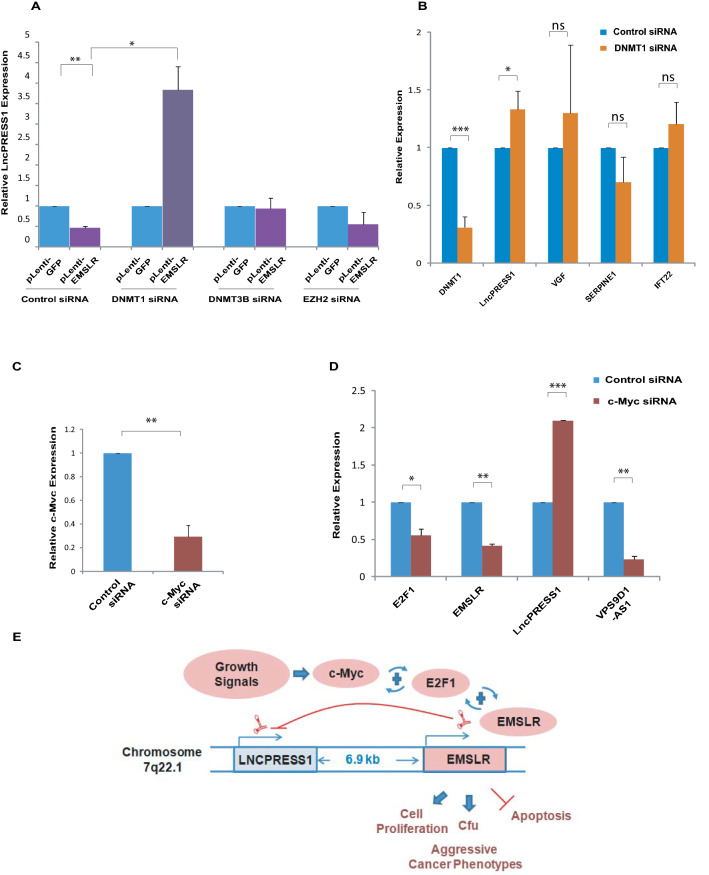
*C-MYC signal transduction pathways control LncPRESS1 expression*. (**A**) The transcriptional repression of *LncPRESS1* mediated by *EMSLR* is dependent on DNA Methyltransferase 1. A549 cells were transduced with lentiviral expressing either control vector or *EMSLR* followed by puromycin selection to obtain stable cells. Control or *EMSLR* stably overexpressing A549 cells were then transfected with control *GL2* siRNA or siRNA targeting *DNMT1*, *DNMT3B* or *EZH2* followed by quantification of *LncPRESS1* levels by individual quantitative real-time PCR with *GAPDH* as an endogenous control. The levels of *LncPRESS1* observed after different siRNA transfections in *EMSLR* expressing stable cells were normalized to non-*EMSLR* expressing cells. (**B**) Effect of *DNMT1* depletion on the expression of genes located in the vicinity of *LncPRESS1*. A549 cells were transfected on three consecutive days with control *GL2* or *DNMT1* siRNA and the levels of indicated genes were quantified by individual quantitative real-time PCR. *GAPDH* was used as the endogenous control for normalization of lncRNA expression. (**C**–**D**), Myc signal transduction pathways control *LncPRESS1* expression. A549 cells were transfected with control *GL2* or siRNA targeting *C-MYC* followed by quantification of *C-MYC*, *E2F1*, *EMSLR*, *LncPRESS1* and VPS9D1-AS1 levels by individual quantitative real-time PCR. *GAPDH* was used as the endogenous control for normalization. The data is represented as mean of two independent experiments ± S.D. *p* values were calculated using two-tailed t-test (*p < 0.05; **p < 0.01, ***p < 0.001, ns, non-significant). (**E**) An illustration depicting that positive feedback loops connect *C-MYC* and *E2F1* signals to cell cycle control*.* The model is based on the following observations: (i) *C-MYC* and *E2F1* activate each other’s transcription^47^, depicted by a ‘ + ’ sign; (ii) *C-MYC* induces *EMSLR* which stabilizes *E2F1* mRNA^23^; (iii) Our observations that *EMSLR* expression is dependent on *E2F1* and *EMSLR* represses *LncPRESS1*. The *LncPRESS1* gene is located 6.9 kb upstream of the *E2F1*-dependent lncRNA, *EMSLR* and both are expressed in the same direction from the cytogenic band 7q22.1. CFU stands for colony-forming units.

Having established that *EMSLR* mediates the transcriptional repression of *LncPRESS1* promoter, we next wanted to identify the mechanism of *EMSLR*-mediated silencing of *LncPRESS1*. It is reported that for transcriptional silencing, lncRNAs recruit chromatin modifiers, such as polycomb repressive complex 2 (PRC2)^42–45^. Ezh2 is the histone methyltransferase subunit of the PRC2 which primarily methylates histone H3 on lysine 27 (i.e. H3K27me3), a mark of transcriptionally silent chromatin. We have observed that overexpression of *EMSLR* downregulated endogenous *LncPRESS1* and thus we reasoned that if in *LncPRESS1* repressed state we deplete the factor mediating that repression, we would observe a derepression of *LncPRESS1* expression. As previously shown, ectopic expression of *EMSLR* led to downregulation of *LncPRESS1* and when we transfected siRNA targeting *EZH2* in *EMSLR* expressing cells we observed that the *LncPRESS1* downregulation caused due to *EMSLR* expression was not affected, implying that *EZH2* is not required for the *EMSLR* mediated suppression of *LncPRESS1* (Fig. 6A). LncRNAs have also been reported to cause gene repression by altering the DNA methylation of target genes: LncRNA Dum recruits DNA Methyltransferase Dnmt1, Dnmt3a, and Dnmt3b to the promoter of DPPA2 gene thereby silencing its expression and stimulating myogenic differentiation^23^. Though *DNMT3B* depletion partially suppressed the *EMSLR*-induced *LncPRESS1* downregulation, a statistically significant effect on *LncPRESS1* levels was not observed. However, silencing of *DNMT1* led to a significant increase In *LncPRESS1* expression, demonstrating that the transcriptional repression of *LncPRESS1* mediated by *EMSLR* is dependent on Dnmt1 (Fig. 6A). Next, we evaluated that effect of *DNMT1* depletion on the genes located in the vicinity of *LncPRESS1*, namely *VGF*, *SERPINE1* and *IFT22*. *DNMT1* depletion led to a moderate but statistically significant increase in *LncPRESS1* levels (Fig. 6B). *VGF*, *IFT22* and *SERPINE1* did display altered expression after *DNMT1* depletion but these changes were not statistically significant. Thus, *LncPRESS1* is moderately upregulated after *DNMT1* downregulation but the neighboring genes do not show a clear pattern. Thus, *DNMT1* depletion by itself lead to only a minor increase in *LncPRESS1* levels but in the presence of overexpressed *EMSLR, DNMT1* depletion causes a significant fourfold increase in *LncPRESS1* levels. Thus, it seems that with decreased DNA methylation due to *DNMT1* depletion, *EMSLR* induces *LncPRESS1*, possibly by independent mechanisms^46^. Though *DNMT1* siRNA depletion led to an almost 70% decrease in *DNMT1* expression, we have not evaluated the decrease in DNA methylation and the hypothesis that decreased DNA methylation may facilitate *EMSLR* induction of *LncPRESS1* needs to be experimentally tested in the future. We have also observed that the expression of *VGF*, *SERPINE 1* and *IFT22* was not significantly altered after *EMSLR* depletion signifying that both *EMSLR* and *DNMT1* specifically regulate *LncPRESS1* while not affecting the other genes in the same genomic region (Fig. 4B).

It has been recently shown that *C-MYC* induces *EMSLR* but neither overexpression nor knockdown of *C-MYC* affected expression of endogenous *LncPRESS1*^23^. When we transfected A549 cells with *C-MYC* siRNA, concurrent with *C-MYC* and *EMSLR* decrease, we observed an increase in *LncPRESS1* levels (Fig. 6C–D). Since the same cell line A549 was used in both studies, we cannot anticipate any reason other than different efficiencies of RNAi depletions. Since *C-MYC* induces the promoter activity of *EMSLR*, its depletion would result in downregulation of *EMSLR* not only because it is a direct target of *C-MYC* but also because, as shown in this study, *EMSLR* expression is dependent on *E2F1*, which is a *C-MYC* target gene^47^. Thus, upregulation of *EMSLR* target *LncPRESS1* after depletion of *C-MYC* exemplifies how *C-MYC* and *E2F1* signal transduction pathways control the network of lncRNA genes to modulate cell proliferation and differentiation (Fig. 6E).

## Discussion

A recent study has reported that *EMSLR* is a direct transcriptional target of oncoprotein *C-MYC*^23^. The authors demonstrated that knockdown of *C-MYC* decreased *EMSLR* expression while overexpression of *C-MYC* induced *EMSLR* expression. It is known that *C-MYC* and *E2F1* can activate each other’s transcription and in conjunction our discovery that *E2F1* induces *EMSLR*, *C-MYC* signal would reach *EMSLR* in two ways^47,48^. First, *C-MYC* would interact with the chromatin fragment comprising the D2 *C-MYC*-binding site within the *EMSLR* promoter. Second, in light of our discovery that *E2F1* induces *EMSLR*, *C-MYC* would indirectly induce *EMSLR* via *E2F1*. The previous study has shown that *EMSLR* cooperates with the RNA binding protein RALY to stabilize *E2F1* mRNA and with our discovery that *E2F1* induces *EMSLR*, it exemplifies the positive feedback loops that amplify the *C-MYC* and the *E2F1* signals during oncogenic transformation.

Like the previous report, we observed that *EMSLR* depletion in A549 cells leads to G1 block and impedes S phase progression inhibiting cell proliferation^23^. One important difference from the previous study is the effect of *C-MYC* overexpression on other lncRNAs in the same locus from where *EMSLR* is expressed (chr7q22.1). Wang et al. reported that neither overexpression nor depletion of *C-MYC* or *EMSLR* affected expression of *LncPRESS1* and IFT22, the neighboring genes of *EMSLR* and thus they claimed a specific effect of *C-MYC* on *EMSLR* expression^23^. However, we observed that depletion of *C-MYC* or *EMSLR* was accompanied by a concurrent increase in *LncPRESS1* levels (Fig. 6C,D). Thus, we propose that *MYC*-*EMSLR*-*LncPRESS1* pathway is functional in cancer cells based on the following results: First, *LncPRESS1* was upregulated after depletion of *EMSLR* (Fig. 4C). Second, overexpression of *EMSLR* led to a significant decrease in the levels of *LncPRESS1*, exemplifying that *EMSLR* represses *LncPRESS1* (Fig. 4E). Next, the depletion of *EMSLR* led to an upregulation of the luciferase activity driven from *LncPRESS1* promoter, signifying that *EMSLR* depletion led to de-repression of *LncPRESS1* promoter (Fig. 5B). Lastly, depletion of *C-MYC* by siRNA was accompanied by a decrease in levels of *EMSLR* and *E2F1* and a concurrent increase in *LncPRESS1* levels (Fig. 6C).

It has been recently shown that *LncPRESS1* sequesters SIRT6, an H3K9ac de-acetylase enhancing the H3K56/K9 acetylation at the pluripotency gene promoters and thus maintaining the pluripotency of stem cells^49^. It was also shown that during differentiation p53 represses *LncPRESS1* resulting in SIRT6-mediated de-acetylation and silencing of pluripotent genes. The previous study was carried out in embryonic stem cells (hESCs) while we have assayed the *EMSLR* effect on *LncPRESS1* in adenocarcinomic human alveolar basal epithelial cells. Whether *EMSLR*-*LncPRESS1* regulation is retained in ESCs needs to be examined but if it is, it would imply that *EMSLR* may be influencing the expression of *LncPRESS1*-dependent pluripotent gene. Whether a gene regulating pluripotency in normal stem cells would then assume an oncogenic function during tumorigenesis is an exhilarating hypothesis to test.

With the advent of sensitive next-generation sequencing technologies thousands of novel RNA transcripts have been discovered over the last two decades. With subsequent understanding of the function of lncRNAs in human diseases, it has become clear that lncRNAs perform vital cellular functions. Despite new lncRNAs being increasingly discovered by high throughput sequencing technologies, only a very small fraction of more than 12,000 annotated lncRNAs genes have been studied in detail. To add to this complexity is that there are multiple transcripts for almost every lncRNA gene with very different final sequences, adding further to the pool of lncRNAs possibly functional in mammalian cells. Moreover, recent studies show that lncRNAs functionally interact with multitude of ncRNA and protein-coding genes forming innumerable regulatory relationships^50^. Thus, functionally evaluating each lncRNA would be an extended process but would unravel the extent of gene networking operational in mammalian cells. Literature is replete with examples of lncRNA regulating protein coding genes but our Pubmed search results returned few examples of one lncRNA gene regulating another lncRNA, as we have shown in this study. Though this would be expected as lncRNA gene has all characteristics of a protein coding gene and would be subject to same regulatory mechanisms, it does add another level of multiplicity to the gene regulatory networks existing in mammalian cells. In summation, in this study we have identified an lncRNA *EMSLR* that maintains the invasive properties of cancer cells and our work exemplifies how *C-MYC* and *E2F1* signal transduction pathways control the network of lncRNA genes to modulate cell proliferation and differentiation. The discovery that oncogenic lncRNA *EMSLR* is dependent on *E2F1* would not only advance our understanding of carcinogenesis but would also present *EMSLR* as a potential target for therapeutic intervention.

## Methods

### Cell culture, cell synchronization and cloning

Experimental procedures have been followed as per previously standardized protocols^51,52^. HEK293T (human embryonic kidney cells with SV40 large T antigen cell line), A549 (adenocarcinomic human alveolar epithelial cell line), H1299, a non-small cell lung cancer (NSCLC) cell line and MRC-5 (human lung fibroblast cell line) cells were maintained in Dulbecco’s modified eagle’s medium (DMEM) supplemented with 10% fetal bovine serum (FBS) along with 1% of 100 units/mL antibiotic and antimycotic solution at 37 °C in a humidified atmosphere with 5% CO_2_ while BEAS-2B (immortalized but a non-tumorigenic lung epithelial cell line) was maintained in 1:1 of F12 and DMEM low glucose medium. For constructing lentiviral vectors expressing *EMSLR*, it was amplified by PCR and cloned into plenti-CMV-puro plasmid. HEK293T cells were transfected with plenti-CMV-puro-*EMSLR* along with helper plasmids expressing packaging vector pMD2.G and envelope vector psPAX2 at a 4:3:1 ratio using Lipofectamine 2000 reagent to generate viral particles. To obtain stable cells expressing *EMSLR*, A549 cells were infected with the lentiviral particles along with 1 µg/ml of polybrene and selected with 1 µg/mL of puromycin 48 h after the infection. For constructing a lentiviral vector to deplete *EMSLR*, a short hairpin RNA (shRNA) that targets *EMSLR* was inserted into AgeI/EcoRI-digested pLKO.1 puro (Addgene). For lentivirus preparation, lentiviral vector pLKO.1 expressing shRNA were co-transfected with packaging vector pMD2.G and envelope vector psPAX2 at a 4:3:1 ratio using Lipofectamine 2000 reagent (Invitrogen) in HEK293T cells. To obtain stable cells expressing shRNA, A549 cells were infected with the lentiviral particles along with 1 µg/ml polybrene and selected with 1 µg/mL of puromycin 48 h after the infection.

### Transfection

For RNAi-mediated gene silencing, small inhibitory RNAs (siRNAs) against *GL2*, *EMSLR*, *EZH2*, *Dnmt3A* and *DNMT1* were custom synthesized by Dharmacon, USA. Cells were transfected with 80 nM of siRNA using Lipofectamine 2000 reagent (Invitrogen) for three consecutive days. The cells were harvested 24 h after the last transfection for flow-cytometric analysis or reverse transcriptase PCR. The sequences used for RNAi are as follows:

GL2: CGUACGCGGAAUACUUCGA;

*EMSLR* shRNA (I): 5′ AAGAGAACGCGGGAUUCAGCC 3′

*EMSLR* siRNA(I): 5′ UAGAGGGAUUCAAGAGACU 3′

*EMSLR* siRNA(II) : 5′ CAGCAAUUCUGGAUAUGGU 3′

*C-MYC* siRNA : 5′ GCUUGUACCUGCAGGAUCU 3′

*E2F1* siRNA : 5′ CCAAGAAGUCCAAGAACCA 3′

*EZH2* siRNA : 5′ GGAUAGAGAAUGUGGGUUU 3′

*DNMT3A* siRNA:—5′ GCAUAAAGGUAGGAAAGUA 3′

*DNMT1* siRNA: 5′GAGAAGAGACGUAGAGUUA 3′

The RT-PCR primers used were as follows (FP, forward primer; RP, reverse primer):

*EMSLR*: FP- GTGCAGATCTCAATGGAAGGA, RP- CAGAAGTCTCTTGAATCCCTCT

*LncPRESS1*: FP- 5′ CAGTAATTCTCCAGCAACAG 3′, RP- 5′ TGGCAGGTAATCATCTCATAT 3′

*DNMT1***:** FP- 5′ ATTATCCGAGGAGGGCTACCTG 3′, RP- 5′ ACTTCTTGCTTGGTTCCCGT 3′

*VGF*: FP- 5′ GACGCGTCCCGATCTTCCC 3′, RP- 5′ CGTTGATCAGCAGAAGGCAGA 3′

*SERPINE1*: FP- 5′ CCCTCTACTTCAACGGCCAG 3′, RP- 5′ GGGCGTGGTGAACTCAGTAT 3′

*IFT22*: FP- 5′ GCCTTGCGAGAGTGGAAAAAC 3′, RP- 5′ GCTGGTAACATGCGGGTTCT 3′

*E2F1*: FP- 5′ GCCAAGAAGTCCAAGAACCAC 3′, RP- 5′ TGGGTCAACCCCTCAAGCC 3′

*ZFAS1*: FP- 5′ GCCATTCGTTCTTTCGCGTC 3′, RP- 5′ TTGGAGGTCCAGTGGTGACT 3′

*SNHG17*: FP- 5′ CCCTGTAAAGTCACGTCCCC 3′, RP- 5′ GGGAAAGCTGGATTGGAGC 3′

*VPS9D1-AS1*: FP- 5′ AAATGAGGCAACGGAAAAGGC 3′, RP- 5′ CCATGCCAAGCTACGGGAA 3′

*PCAT6*: FP- 5′ GCCTTCGCCCCTAGATACAC 3′, RP- 5′ GGAAGGGTGGTGGTAGAAGC 3′

*LINC00467*: FP- 5′ ACAGGTTGTTTCTCTGCAGTTT 3′, RP- 5′ ATCTATGTCGGGATCGGTGCTG 3′

*CCNA2*: FP- 5′ GGACCAGGAGAATATCAACCCG 3′, RP- 5′ AAGGGGTGCAACCCGTCTC 3′

*CDC45*: FP- 5′ ATCATGGGACATCGTCAGCC 3′, RP- 5′ TGCACCCACTGGTCTGTTAG 3′

*CDK2*: FP- 5′ CCTGAAATCCTCCTGGGCTG 3′, RP- 5′ CCCAGAGTCCGAAAGATCCG 3′

Primers for cloning of *LncPRESS1* promoter in pGL4.20:

FP- 5′CGGCTAGCCCACATTAATTTTCCGTGAAAAAATCTGTCAGTGGCAC 3′,

RP- 5′ CCGCTCGAGCTACCAGGCCATCTTGAGCCTGT 3′

Primers for cloning *EMSLR* in pLenti-GFP:

FP- 5′ CGCGGATCCGTTTCCACCTAGGACTACAGGCTC 3′

RP- 5′TTATGCGGCCGCTATGGCCGACGTCGACTTTCATTTCACCTTTAATGATTATTCAAGAC 3′

### Luciferase reporter assay

The firefly luciferase-encoding reporter plasmids pGL4.20 [luc2] and pRL-TK were obtained from Promega (Madison, WI, USA). The pRL-TK which encodes renilla luciferase was used as an internal control for transfection efficiency. The − 1,500 bp to + 50 bp upstream region of *LncPRESS1* transcription start site was cloned into pGL4.20. Control or *EMSLR*-depleted or *EMSLR* overexpressing A549 cells were co-transfected with pGL4.20-*LncPRESS1* and pRL-TK and 24 h later the cells were lysed and firefly and renilla luciferase luminescence were sequentially measured according to the manufacturer’s protocol. The firefly luciferase activity was normalized to renilla luciferase activity.

### Cell cycle analysis and flow cytometry

Cell cycle analysis and flow cytometry were carried out as per previously standardized protocols^51,52^. For cell cycle analysis, the cells were harvested and fixed with 70% ethanol at 4 °C for 1 h. Following fixation, the cells were washed with 1X PBS and the cell pellet was resuspended in 1X PBS with 0.1% Triton X- 100, 20 mg/mL RNase A and 70 mg/mL propidium iodide and then the stained cells were analyzed by flow cytometry. For arresting the cells at G2/M transition, the cells were incubated with nocodazole (100 ng/ml) for 16 h before harvesting and fixation with 70% ethanol. The flow cytometry data was acquired on Becton Dickinson FACS Canto machine using BD FACS Diva software. Cell cycle distribution was evaluated by Dean/Jett/Fox method using the FlowJo software. To study the BrdU (5-bromo-2-deoxyuridine) incorporation, cells were cultured in medium containing 100 μM BrdU (BD Biosciences) for 30 min, prior to harvesting. After fixation, cells were treated with 2 N HCl for 15–20 min for denaturing the DNA, followed by a neutralization step of 5 min at room temperature with 0.1 M sodium tetraborate (pH 8.5). Cells were then washed with a blocking solution comprising of 3% bovine serum albumin (BSA) in PBS containing 0.1% Triton X-100 followed by incubation with mouse anti-BrdU antibody (dilution 1:10 in blocking solution) conjugated to Fluorescein isothiocyanate (FITC) for 1 h. After antibody staining, cells were washed with 1X PBS and DNA was stained with propidium iodide and run on FACS machine as previously described. For Apoptosis detection control *GL2* siRNA or *EMSLR* siRNA transfected cells were detached using Accutase enzyme and FACS was performed using FITC- Annexin V Apoptosis Detection Kit (BioLegend's) was used according to the manufacture instructions.

### RNA extraction and quantitative real-time PCR

RNA extraction and quantitative real-time PCR were carried out as per previously standardized protocols^51,52^. Total RNA was extracted from cells using TRIzol reagent (Takara Biosciences) and reverse transcribed into cDNA using Moloney murine leukemia virus reverse transcriptase (Invitrogen). The qRT-PCR reactions were carried out in duplicates in 10 μL volume for the expression analysis. The reaction mixture contained SYBR Select master mix (2X, Takara Biosciences), cDNA template and forward and reverse gene or lncRNA specific primers (0.1 μM each). Target sequence amplification temperature profile followed was as follows: Initial denaturation for 10 min at 95 °C, followed by 40 cycles of 10 s at 95 °C and amplification for 30 s at annealing temperature of 60 °C. Finally, a melt curve analysis was carried out at a temperature range of 60 °C to 95 °C for 20 min. The *GAPDH* was used as internal control for lncRNA and mRNA quantification. Results were calculated using ΔΔCt method to determine the fold change in expression between the experimental and control groups.

### Cell proliferation and clonogenic assays

Cell proliferation and clonogenic assays were carried out as per previously standardized protocols^51,52^. For MTT cell proliferation assay, thirty thousand A549 cells were seeded in triplicates in 96-well cell culture dishes with 500 μl media per well. The MTT substrate, thiazolyl blue tetrazolium bromide was added to cells in culture at a final concentration of 0.5 mg/ml and incubated at 37 °C. After 3–4 h the cells were resuspended in 500 μl of dimethyl sulfoxide (DMSO) and shaken for 15 min. The quantity of formazan was measured by recording changes in absorbance at 570 nm and 630 nm (reference wavelength) using a microplate reader (BioTekPowerWave XS). For cell viability count, trypan blue exclusion method was utilized where *EMSLR*-depleted or control A549 cells were collected and dissolved in 1 ml of 1X PBS and 20 μl of cell suspension was stained with an equal volume of 0.4% trypan blue. Viable cells, which excluded trypan blue dye, were counted in quadruplicate using a glass haemocytometer. For clonogenic assay, *EMSLR*-depleted or control A549 cells were counted and 1,000 cells were seeded in a 6-well culture dish in triplicates. After 12 days of incubation, plates were gently washed with 1X PBS and stained with 0.1% crystal violet. Colonies with over 50 cells were manually counted.

### Data collection

LncRNAs expression from LUAD and LUSC was downloaded from TANRIC (the Atlas of Noncoding RNAs in Cancer) platform (https://ibl.mdanderson.org/tanric/_design/basic/index.html)^32^. All of these samples analyzed were from the Cancer Genomic Atlas (TCGA, https://cancergenome.nih.gov/). For LUAD, transcriptional profiles for 488 tumor and 58 normal samples were downloaded while for LUSC transcriptional profiles for 220 tumor and 17 normal samples were downloaded^32^. The average FPKM values of individual lncRNAs in tumor and normal samples were compared to identify upregulated or downregulated lncRNAs in each cancer. A fold change value of greater than two indicated that the expression of the gene is upregulated compared with the normal and the tumor samples, whereas a fold change of less than 0.5 indicated downregulated expression in tumor samples. The accession numbers for *EMSLR* and *LncPRESS1* are ENSG00000232445 and ENSG00000232301, respectively. Kaplan–Meier estimates of the survival of patients with low or high levels of expression of *EMSLR* and *LncPRESS1* were done on GEPIA (Gene Expression Profiling Interactive Analysis) platform. Statistical Analysis: The results were presented as mean ± standard deviation (SD) and analyzed with Student’s t test. P-value of less than 0.05 was considered significant, unless noted otherwise. All methods were performed in accordance with the relevant guidelines and regulations as explained in the editorial and publishing policies of Scientific Reports.
